# *Lactobacillus acidophilus* and propionate attenuate Sjögren’s syndrome by modulating the STIM1-STING signaling pathway

**DOI:** 10.1186/s12964-023-01141-0

**Published:** 2023-06-14

**Authors:** Jin Seok Woo, Sun-Hee Hwang, SeungCheon Yang, Kun Hee Lee, Yeon Su Lee, Jeong Won Choi, Jin-Sil Park, JooYeon Jhun, Sung-Hwan Park, Mi-La Cho

**Affiliations:** 1 Rheumatism Research Center, Catholic Research Institute of Medical Science, College of Medicine, The Catholic University of Korea, Seoul, 06591 Korea; 2 Lab of Translational ImmunoMedicine, Catholic Research Institute of Medical Science, College of Medicine, The Catholic University of Korea, Seoul, 06591 Korea; 3 Department of Biomedicine & Health Sciences, College of Medicine, The Catholic University of Korea, Seoul, 06591 Korea; 4 Division of Rheumatology, Department of Internal Medicine, Seoul St. Mary’s Hospital, College of Medicine, The Catholic University of Korea, Seoul, 06591 Korea; 5 Department of Medical Life Sciences, College of Medicine, The Catholic University of Korea, Seoul, 06591 Korea

**Keywords:** Sjögren’s syndrome, *Lactobacillus acidophilus*, Propionate, Stromal interaction moleculae 1 (STIM1), Stimulation of interferon genes (STING)

## Abstract

**Background:**

Sjögren’s syndrome (SS) is an autoimmune disease characterized by inflammation of the exocrine gland. An imbalance of gut microbiota has been linked to SS. However, the molecular mechanism is unclear. We investigated the effects of *Lactobacillus acidophilus* (*L*. *acidophilus*) and propionate on the development and progression of SS in mouse model.

**Methods:**

We compared the gut microbiomes of young and old mice. We administered *L*. *acidophilus* and propionate up to 24 weeks. The saliva flow rate and the histopathology of the salivary glands were investigated, and the effects of propionate on the STIM1-STING signaling pathway were evaluated in vitro.

**Results:**

Lactobacillaceae and *Lactobacillus* were decreased in aged mice. SS symptoms were ameliorated by *L*. *acidophilus*. The abundance of propionate-producing bacterial was increased by *L*. *acidophilus*. Propionate ameliorated the development and progression of SS by inhibiting the STIM1-STING signaling pathway.

**Conclusions:**

The findings suggest that *Lactobacillus acidophilus* and propionate have therapeutic potential for SS.

Video Abstract

**Supplementary Information:**

The online version contains supplementary material available at 10.1186/s12964-023-01141-0.

## Background

Sjögren’s syndrome (SS), first described in 1933 [1], is an autoimmune disease mediated by the infiltration of lymphocytes into the exocrine glands, including the salivary and lacrimal glands [2, 3]. Lymphocyte infiltration leads to destruction of glandular tissue and dysfunctional glandular secretion, followed by dry eyes and mouth [4, 5]. Although SS does not affect life expectancy, it reduces the quality of life. As with other autoimmune diseases, there is no cure for SS, and treatment focuses on relieving symptoms.

Recent studies have revealed dysbiosis of the microbiome in animal models of, and patients with, autoimmune diseases, including SS [6, 7]. Moon et al. reported that the phylum *Bacteroidetes* and the genus *Bifidobacterium* were increased, whereas the *Firmicutes:Bacteroidetes* ratio, an indicator of gut dysbiosis, was decreased, in patients with SS. Cano-Ortiz et al. reported lower diversity and richness of gut microbiota in patients with SS [8]. Supplementation with probiotics can enhance gut-associated immunity [9, 10]. *Lactobacillus acidophilus*, a common probiotic [11, 12], is a candidate immunomodulator [13, 14]. *L*. *acidophilus* attenuates intestinal inflammation by inhibiting endoplasmic reticulum (ER) stress [15] and modulating the Th17:Treg balance [16]. In addition, in one study, *L*. *acidophilus* supplementation had a therapeutic effect in lupus-prone mice by regulating the specific intracellular adhesion molecule-3 grabbing non-integrin homolog-related 3 (SIGNR3) pathway [17]. SIGNR3 is a critical mediator of the interaction between *L*. *acidophilus* and host immune cells [17–19].

Bacteria produce metabolites, including vitamins and short-chain fatty acids (SCFAs), from dietary substrates [20, 21]. SCFAs reportedly have a therapeutic effect in autoimmune diseases [22, 23]. Wen et al. showed that concentrations of SCFAs—including acetate, propionate, butyrate, isobutyrate, valerate, and isovalerate—were markedly decreased in mice with DSS-induced colitis, and butyrate regulated the Treg:Th17 balance [24]. We reported that butyrate ameliorated SS by inducing interleukin-10 (IL-10)-producing B cells [7] and attenuated rheumatoid inflammation in an animal model of rheumatoid arthritis [25]. Propionate is produced by the gut microbiota, including *Ruminococcaceae*, *Clostridiaceae*, and *Prevotellaceae* [26–28]. Tedelind et al. showed that propionate has anti-inflammatory effects on inflammatory bowel diseases [29]. Furthermore, Filippone et al. reported that propionate has anti-inflammatory and antioxidant effects [30].

Type I interferon (IFN) has antiviral activity and is induced by stimulation of interferon genes (STING) and the ER signaling adaptor and suppressed by stromal interaction molecule 1 (STIM1), an ER Ca^2+^ sensor [31, 32]. The type I IFN pathway is involved in the onset of autoimmune diseases, such as SS, systemic lupus erythematosus (SLE), systemic sclerosis, and RA [33, 34]. Type I IFN stimulates B cells to produce autoantibodies [35, 36], a hallmark of autoimmune diseases. It shows potential as a therapeutic target for autoimmune diseases [37, 38].

The roles of microbiota/SCFAs in type I IFN production is controversial. Villena et al. reported that *Lactobacillus rhamnosus* induces type I IFN production [39]. Also, Yang et al. reported that microbiota-derived butyrate suppresses type I IFN [40], whereas acetate lead to increase type I IFN production [41]. However, the effect of L. acidophilus and propionate in type I IFN production has not been studied. In this study, we evaluated the role of *L. acidophilus* and propionate in the development and progression of SS and their therapeutic potential.

## Methods

### Animals

We purchased female NOD/ShiLtJ mice from Jackson Laboratories (Bar Harbor, ME, USA). The mice were housed under specific-pathogen-free conditions at the Catholic Research Institute of Medical Science, The Catholic University of Korea, and were fed a diet sterilized by gamma ray (TD 2018S; Harlan Laboratories, Tampa, FL, USA) and autoclaved water.

#### Gut microbiome analysis

Mice with average pain data and histopathology results for each group were analyzed. Samples were analyzed in Chun-lab; results are available at http://www.ezbiocloud.net/apps. The cecal microbiome was analyzed to the family and genus levels.

### Measurement of salivary secretion in NOD/ShiLtJ mice

Mice were anesthetized by inhalation of isoflurane (2%). Whole saliva was collected for 7 min from the oral cavity, starting 90 s after intraperitoneal injection of pilocarpine (100 µg/mouse; Sigma-Aldrich, St. Louis, MO, USA). Saliva flow rates were expressed as microliters of saliva secreted per gram body weight per min (µL/g/min).

### Supplementation with *L. acidophilus* or propionate

*L. acidophilus* was purchased from CNS Pharm Korea (Jincheon, Korea). *L. acidophilus* was resuspended in saline a concentration of 125 mg/mL (2 × 10^11^ CFU/mL) and heat killing at 80 °C for 30 min. Twelve-week-old NOD mice were orally administered 50 mg/kg of *L. acidophilus* daily for 12 weeks in saline (*N* = 10) or saline alone (*N* = 15). Twelve-week-old NOD mice were intraperitoneally administered 200 mg/kg sodium propionate (Sigma) in saline (*N* = 4) three times per week for 10 weeks or saline alone (*N* = 5).

### Histopathological analysis

Tissue was fixed in 10% formalin and embedded in paraffin. Sections were stained with hematoxylin and eosin (H&E). Inflammation of the salivary gland was scored as described previously [42]. The scoring criteria were as follows: score 0, no infiltrate; score 1–1.5, 1–2 foci per section; score 2–2.5, 3–5 foci per section; score 3, 6–10 foci per section; and score 4, > 10 foci per section.

### Immunohistochemistry

Immunohistochemistry was performed with a Vectastain ABC Kit (Vector Laboratories, Burlingame, CA, USA). Tissue sections were incubated with anti-IL-6, -IL-17, -TNF-α, -STIM1, or -phospho-STING primary antibodies at 4 °C overnight followed by the appropriate biotinylated secondary antibody. Next sections were incubated with streptavidin–peroxidase complex for 1 h. DAB chromogen (Dako, Carpinteria, CA, USA) was added as the substrate. Stained cells were visualized by microscopy (Olympus, Center Valley, PA, USA). Antibodies are listed in Supplementary Table 1.

### Confocal microscopy

Salivary gland tissue was stained with anti-CD4 and -IL-17 antibodies for Th17 cells and anti-CD4, -CD25, and -FoxP3 antibodies for Treg cells at 4 °C overnight. Secondary antibodies conjugated with FITC, APC, and PE were incubated at room temperature for 2 h. Nuclei were stained with 4,’6-diamidino-2-phenylindole. Confocal images were obtained with an LSM 700 confocal microscope (Zeiss, Oberkochen, Germany) at 200 × magnification. Antibodies are listed in Supplementary Table 1.

### Cell culture

Human submandibular gland (HSG) cells were cultured with recombinant human TNF-α (2 ng/mL) and human IL-17 (20 ng/mL) in the absence or presence of *L*. *acidophilus* (10 or 100 µg/mL) for 48 h. Mouse splenocytes were cultured with anti-CD3 (0.5 µg/mL) in the absence or presence of *L*. *acidophilus* (10 or 100 µg/mL) or propionate (0.2 or 1 mM) for 48 h. Mouse non-T cells from spleen were cultured with LPS (100 ng/mL) in the absence or presence of *L*. *acidophilus* (10 or 100 µg/mL) for 48 h.

### mRNA isolation, cDNA synthesis, and real-time quantitative PCR

mRNA was extracted with TRI Reagent (Molecular Research Center, Cincinnati, OH, USA) according to the manufacturer’s instructions. PCR amplification was performed with an Applied Biosystems StepOne Plus Real-Time PCR System (Applied Biosystems, Foster City, CA, USA) and SensiFAST SYBR Hi-ROX (Bioline USA, Taunton, MA, USA) according to the manufacturer’s instructions. Expression was normalized to that of β-actin. Primer sequences are listed in Supplementary Table 2.

### Intracellular staining and flow cytometry

Cells were isolated from the spleen and salivary glands and stimulated with 25 ng/mL phorbol myristate acetate and 250 ng/mL ionomycin (Sigma-Aldrich) in the presence of GolgiStop (BD Biosciences, San Jose, CA, USA) for 4 h. Cells were reacted with anti-CD4, -CD19, or -CD11c antibodies at 4 °C for 30 min; permeabilized with Cytofix/Cytoperm solution (BD Pharmingen, Franklin Lakes, NJ, USA); and stained intracellularly with anti-IFN-γ, -IL-4, -IL-17, or -IFN-α antibodies. Samples were analyzed with a FACS Calibur (BD Pharmingen) fluorescence-activated cell sorting (FACS) instrument, and data were analyzed with FlowJo (Tree Star, Ashland, OR, USA). Antibodies are listed in Supplementary Table 1.

### Ethics approval and consent to participate

Animal procedures were performed in accordance with the Laboratory Animals Welfare Act, the Guide for the Care and Use of Laboratory Animals, and the Guidelines and Policies for Rodent Experiments of the Institutional Animal Care and Use Committee of the School of Medicine, The Catholic University of Korea (no. 2021-0277-02).

### Statistical analysis

Data are means ± standard deviation (SD). Statistical analyses were performed in Prism version 5 for Windows (GraphPad Software, San Diego, CA, USA). Normally distributed continuous data were analyzed by parametric Student’s *t* test. Differences in means among groups were subjected to one-way analysis of variance (ANOVA). *p* < 0.05 was taken to indicate statistical significance.

## Results

### Dysbiosis of the gut microbiome

**Fig. 1:**
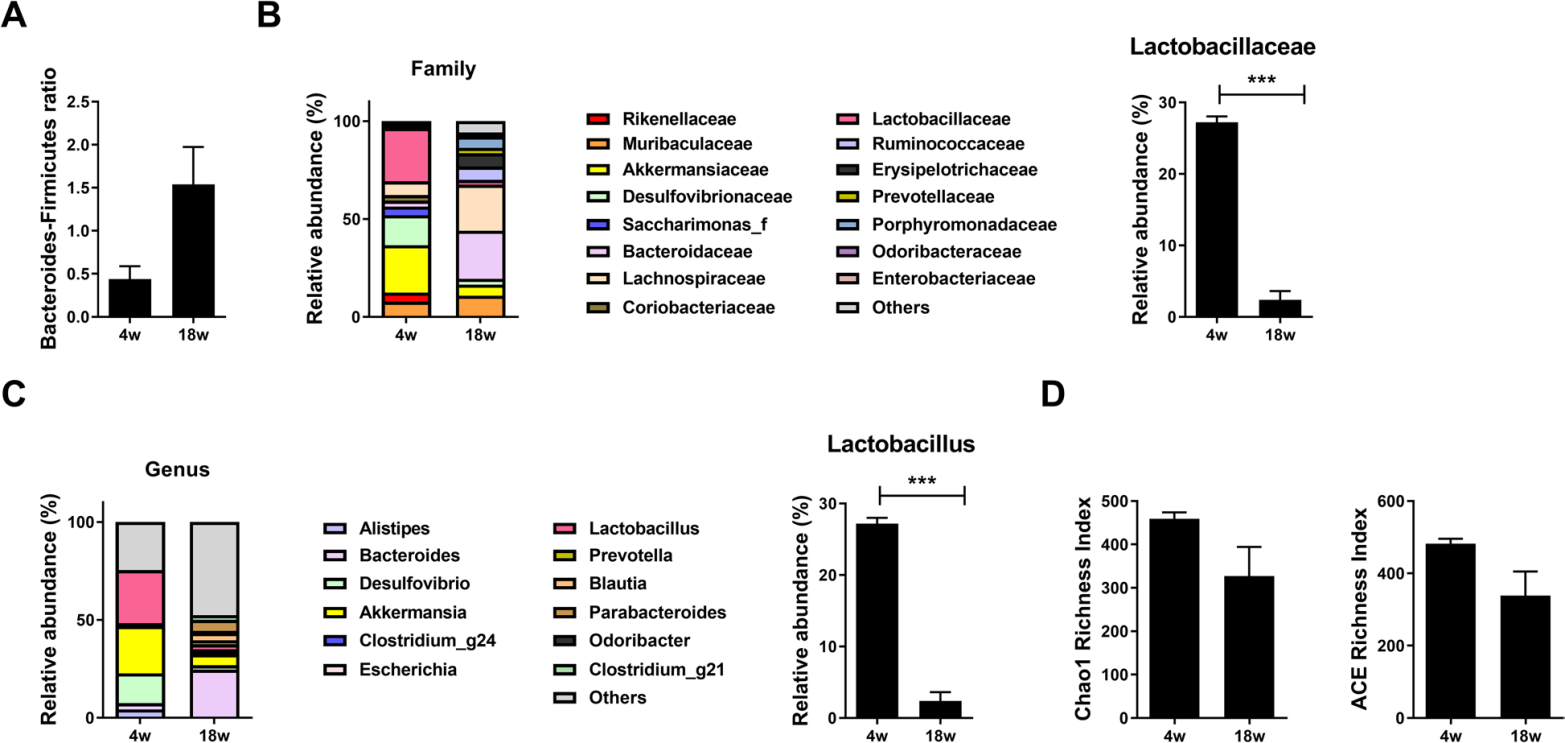
Changes in the microbiome with age. Cecal microbiome analyses of 4- and 18-week-old NOD mice. **A** *Bacteroides:Firmicutes* ratio in 4- and 18-week-old NOD mice. **B** Abundance (family level) of the gut microbiome (left) and *Lactobacillaceae* (right) in 4- and 18-week-old NOD mice. **C** Abundance (genus level) of the gut microbiome (left) and *Lactobacillus* (right) in 4- and 18-week-old NOD mice. **D** Chao1 and ACE indices. Data are means ± SD (*p < 0.05, **p < 0.005, ***p < 0.001)

NOD/ShiLtJ mice spontaneously develop inflammatory lesions in the salivary gland at 10 weeks old. Therefore, we examined the cecal content of young (4-week-old) and old (18-week-old) NOD/ShiLtJ mice. The *Bacteroides:Firmicutes* ratio was increased in old mice (Fig. 1A), and the gut microbiota differed markedly at the family level between young and old mice (Fig. 1B). The abundance of *Lactobacillaceae* was markedly decreased in old mice (Fig. 1B). The gut microbiota also differed at the genus level (Fig. 1C). The Chao1 and ACE indices suggested lower species richness in the gut microbiomes of old mice compared to young mice (Fig. 1D). These data suggest that dysbiosis of the gut microbiome is related to SS.

### *L. acidophilus* attenuates SS and the infiltration of lymphocytes into the salivary gland

**Fig. 2:**
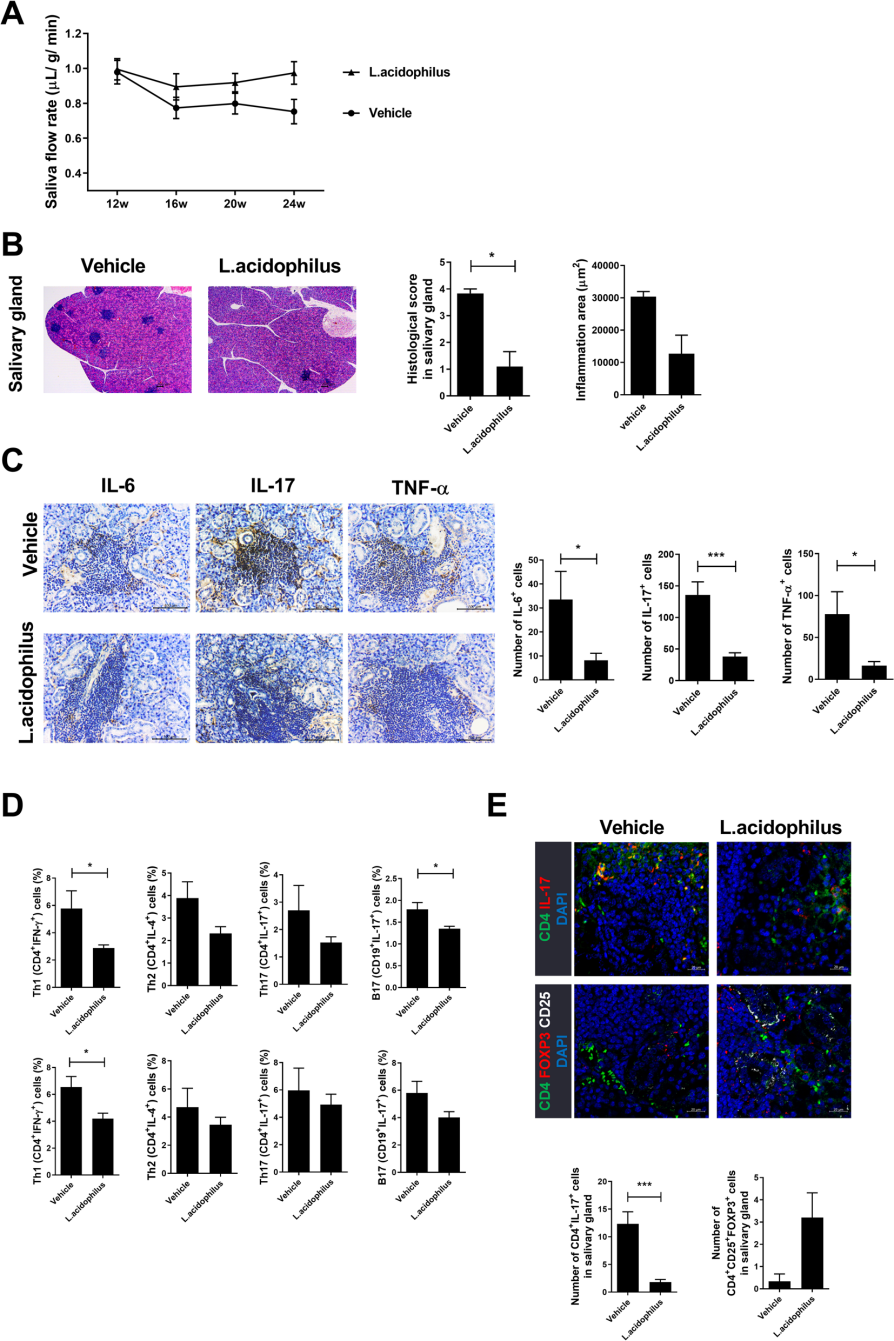
Therapeutic effects of *L*. *acidophilus* in SS. *L*. *acidophilus* was administered orally to NOD mice, which were monitored for 24 weeks. Salivary glands and the spleen were harvested. **A** Saliva flow rate at 12, 16, 20, and 24 weeks. **B** Salivary glands were stained with H&E. The histological score (left) and inflammation area (right) are shown. **C** Salivary gland tissues were stained for IL-6, IL-17, and TNF-α. Numbers of cells positive for IL-6 (left), IL-17 (center), and TNF-α (right) are shown. **D** Splenocytes (top) and salivary glands (bottom) analyzed by flow cytometry for percentages of Th1 (CD4^+^IFN-γ^+^), Th2 (CD4^+^IL-4^+^), Th17 (CD4^+^IL-17^+^), and B17 (CD19^+^IL-17^+^) cells. **E** Salivary glands were analyzed by confocal microscopy. Representative images of Th17 (CD4^+^IL-17^+^; top) and Treg (CD4^+^CD25^+^FOXP3.^+^; bottom) cells are shown. Bar graphs show the numbers of cells positive for Th17 and Treg. Data are means ± SD (*p < 0.05, **p < 0.005, ***p < 0.001)

To determine the role of *Lactobacillus* in SS, we administered *L*. *acidophilus* to 12-week-old NOD/ShiLtJ mice. *L*. *acidophilus* improved the saliva flow rate and tissue inflammation (Fig. 2A, B). Furthermore, the infiltration of inflammatory cytokine (such as IL-6, IL-17, and TNF-α)-producing cells into the salivary gland was decreased by *L*. *acidophilus* (Fig. 2C), and infiltration of Th1, Th2, Th17, and B17 cells into salivary gland and spleen was also decreased by *L*. *acidophilus* (Fig. 2D, E), whereas Treg cells were increased by *L*. *acidophilus.* Our results show that *L*. *acidophilus* has immunomodulatory (anti-inflammatory) effects in SS.

### *L. acidophilus* induces the SIGNR3 pathway

**Fig. 3:**
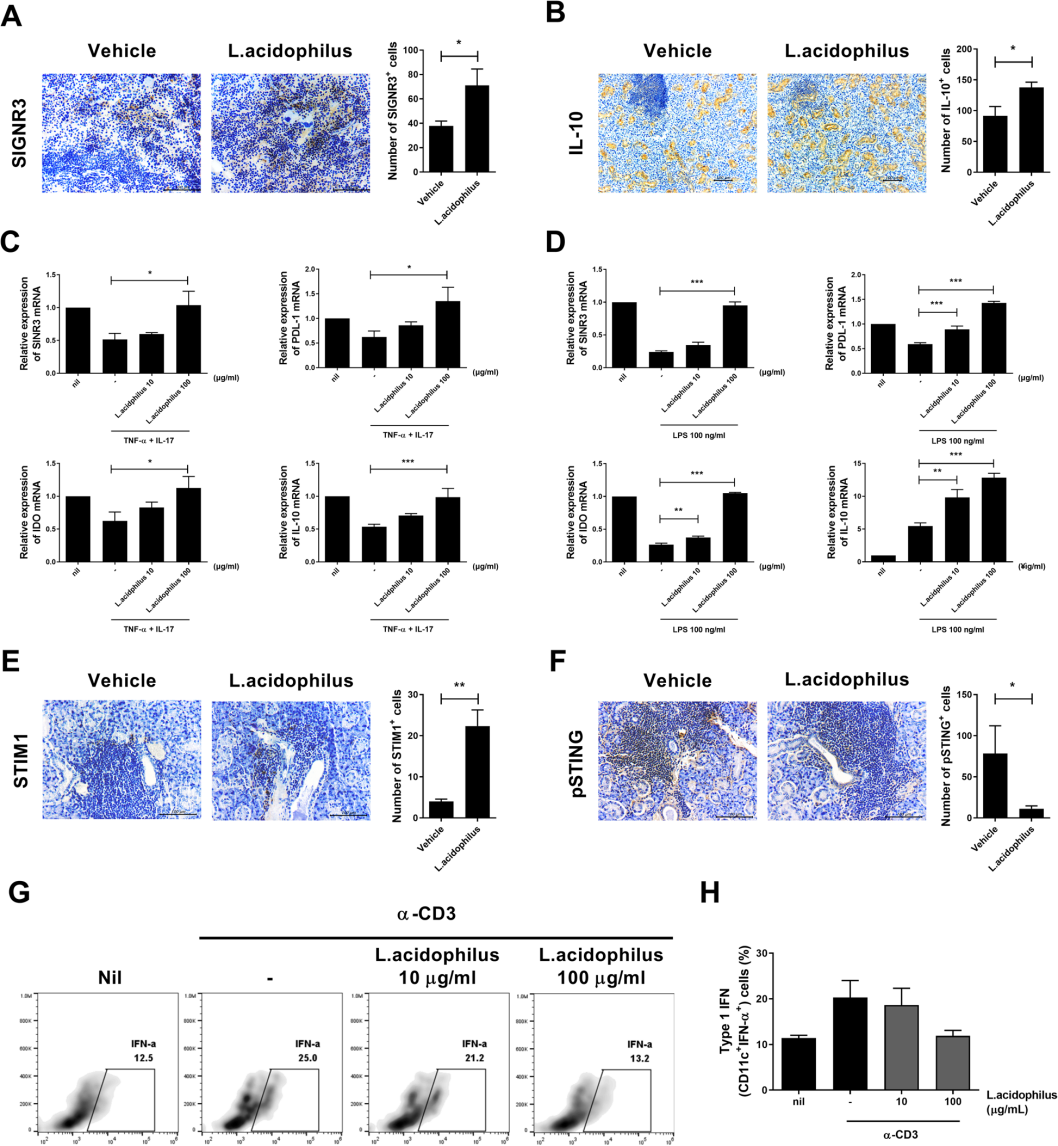
Regulation of SINGR3 expression by *L*. *acidophilus*. Salivary glands were harvested from the vehicle and *L*. *acidophilus* groups. **A** Representative images of SINGR3-positive cells in salivary glands and average numbers of SINGR3-positive cells. **B** Representative images of IL-10-positive cells in salivary glands and average numbers of IL-10-positive cells. **C** HSG cells were stimulated with TNF-α (2 ng/mL) and IL-17 (20 ng/mL) in the absence or presence of *L*. *acidophilus* (10 or 100 µg/mL) for 48 h and harvested for mRNA extraction and real-time PCR. Bar graphs show mRNA levels of SINGR3 (top and left), PD-L1 (top and right), IDO (bottom and left), and IL-10 (bottom and right). **D** Mouse non-T cells from the spleen were stimulated with LPS (100 ng/mL) in the absence or presence of *L*. *acidophilus* (10 or 100 µg/mL) for 48 h and harvested for mRNA extraction and real-time PCR. mRNA levels of SINGR3 (top and left), PD-L1 (top and right), IDO (bottom and left), and IL-10 (bottom and right) are shown. **E** Representative images of STIM1 in the salivary gland and average numbers of STIM1-positive cells. **F** Representative images of phosphor-STING in the salivary gland and average numbers of phosphor-STING-positive cells. **G** Splenocytes were stimulated with anti-CD3 (0.5 µg/mL) in the absence or presence of *L*. *acidophilus* (10 or 100 µg/mL) for 48 h and harvested for flow cytometry. FACS plots show percentages of type I IFN–producing dendritic cells. **H** Bar graphs show average percentages of type I IFN–positive cells. Data are means ± SD (*p < 0.05, ***p < 0.001)

SIGNR3 regulates intestinal immunity [19]. Expression of SIGNR3 and the number of IL-10-producing cells in the salivary gland were increased by *L*. *acidophilus* (Fig. 3A, B), as were mRNA levels of SINGR3, PD-L1, IDO, and IL-10 (Fig. 3C, D). These data suggest that *L*. *acidophilus* ameliorates SS by enhancing immunomodulation of the SINGR3 pathway.

### *L. acidophilus* attenuates SS by inhibiting the STIM1-STING pathway

STING induces type I IFN, a key mediator of autoimmune diseases, including SS. To investigate the effects of *L*. *acidophilus* on the STING pathway, we measured the expression of STIM1, a negative regulator of STING, and phospho-STING in salivary glands. STIM1 and phospho-STING expression were increased and decreased, respectively, by *L*. *acidophilus* (Fig. 3E, F). In addition, *L*. *acidophilus* decreased the number of type I IFN–producing cells (Fig. 3G, H). These data suggest that *L*. *acidophilus* ameliorates SS by inhibiting the production of type I IFN.

### Effects of *L. acidophilus* on the gut microbiome

**Fig. 4:**
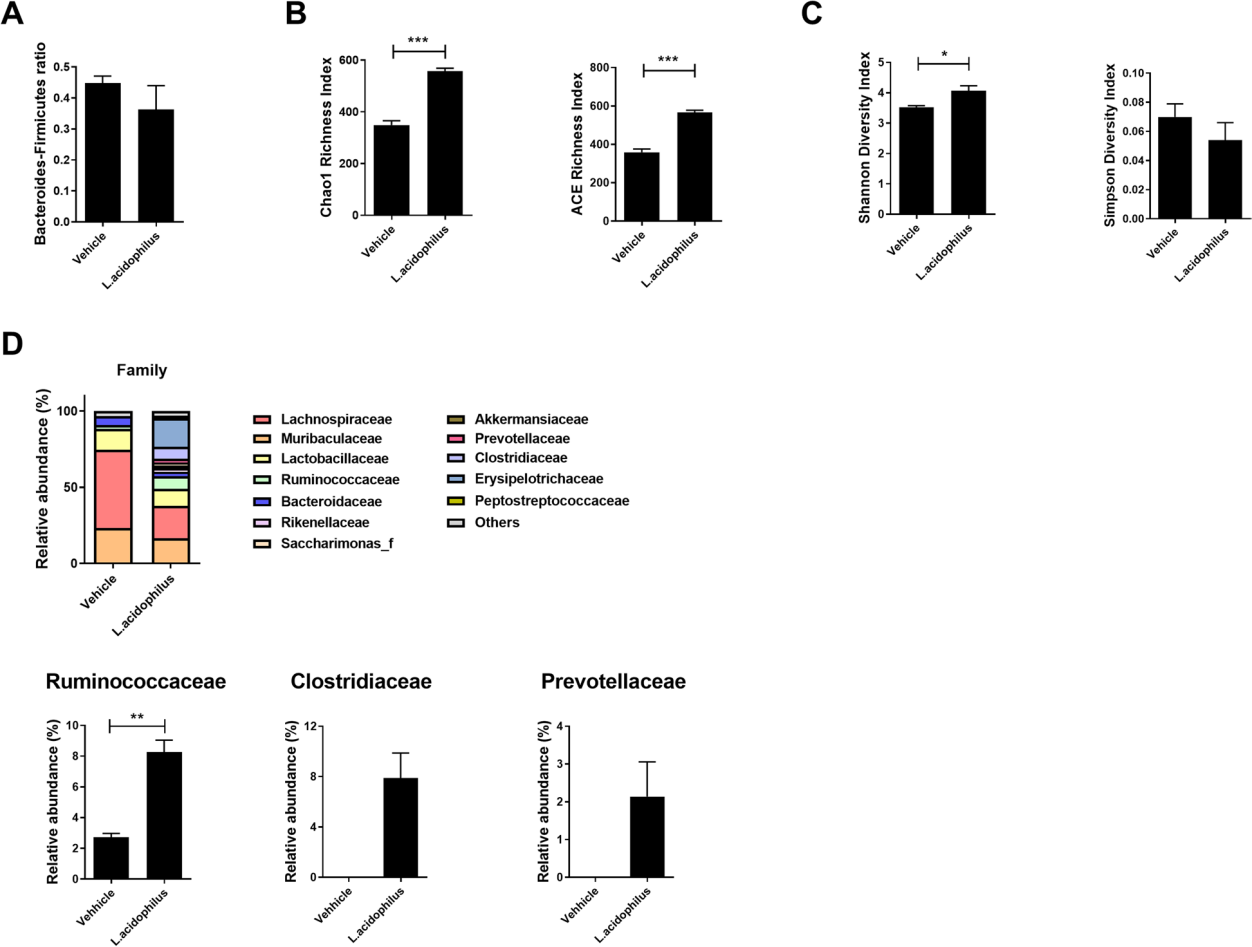
*L*. *acidophilus* affects the gut microbiota. Cecal microbiome analyses of the vehicle and *L*. *acidophilus* groups. **A** *Bacteroides:Firmicutes* ratio. **B** Chao1 and ACE indices. **C** Shannon and Simpson diversity indices. **D** Composition of gut microbiota at the family level (top). Bar graphs at the bottom show the abundance of *Ruminococcaceae* (left), *Clostridiaceae* (center), and *Prevotellaceae* (right). Data are means ± SD (*p < 0.05, **p < 0.005, ***p < 0.001)

The *Bacteroides:Firmicutes* ratio and species richness and diversity were improved by *L*. *acidophilus* (Fig. 4A-C)). The abundance of propionate-producing bacteria, such as *Ruminococcaceae*, *Clostridiaceae*, and *Prevotellaceae*, was significantly increased by *L*. *acidophilus* (Fig. 4D). These data suggest that *L*. *acidophilus* ameliorates SS by altering the gut microbiome.

### Propionate attenuates SS and the infiltration of lymphocytes into the salivary gland

**Fig. 5:**
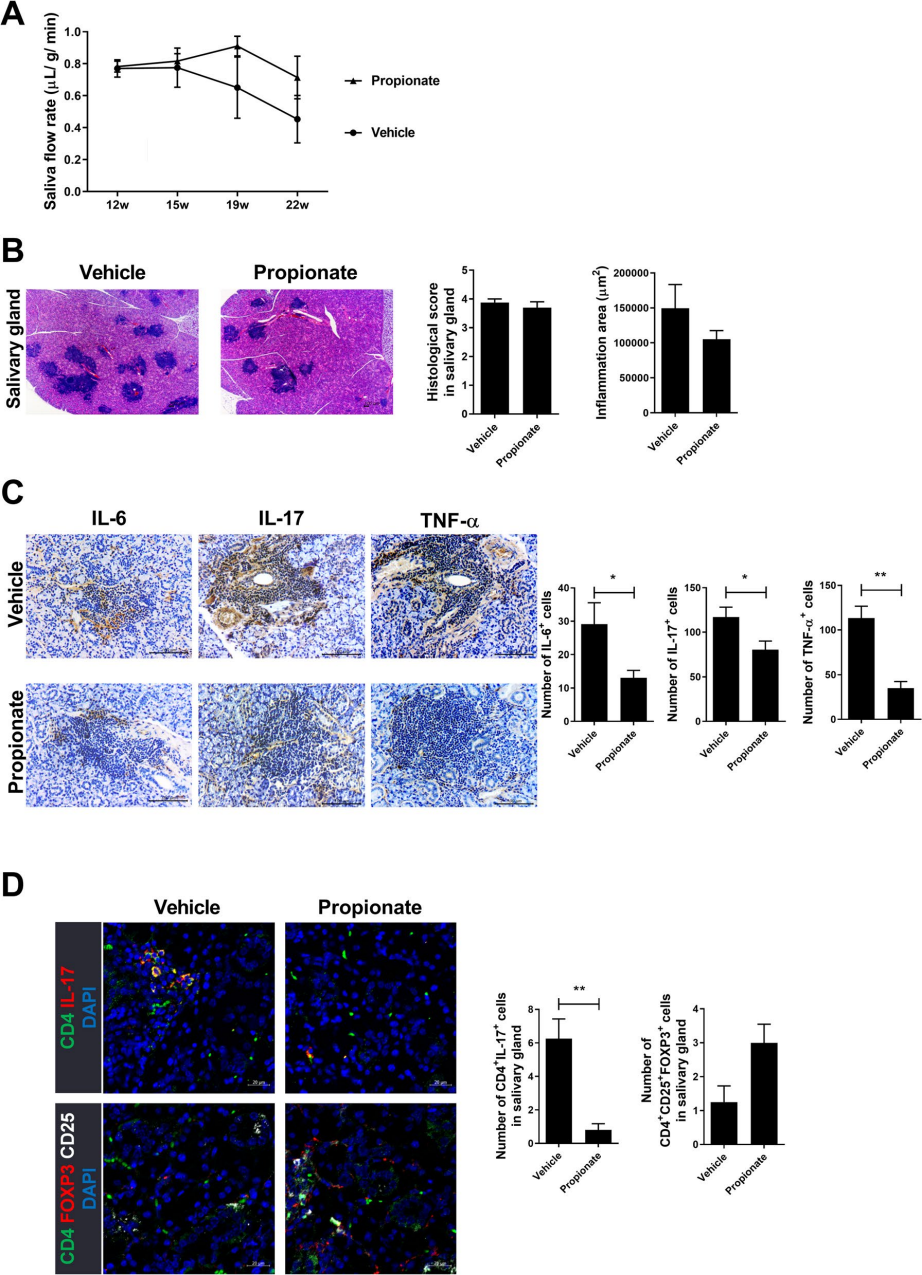
Therapeutic effects of propionate in SS. Propionate (200 mg/kg) was administered intraperitoneally to 12-week-old NOD mice, which were monitored for 24 weeks. Salivary glands and the spleen were harvested. **A** Saliva flow rate at 12, 16, 19, and 23 weeks. **B** Salivary glands were stained with H&E. Bar graphs show the histological score (left) and inflammation area (right). **C** Salivary gland tissue stained for IL-6, IL-17, and TNF-α and numbers of cells positive for IL-6 (left), IL-17 (center), and TNF-α (right). **D** Salivary glands were visualized by confocal microscopy. Representative images of Th17 (CD4^+^IL-17^+^; top) and Treg (CD4^+^CD25^+^FOXP3.^+^; bottom) cells are shown. Bar graphs show numbers of Th17- and Treg-positive cells. Data are means ± SD (*p < 0.05, **p < 0.005, ***p < 0.001)

To investigate the role of propionate in SS, we administered propionate to 12-week-old NOD/ShiLtJ mice. Propionate improved the saliva flow rate and tissue inflammation (Fig. 5A, B). Furthermore, it decreased the infiltration of inflammatory cytokine (IL-6, IL-17, and TNF-α)-producing cells into the salivary gland (Fig. 5C). The Th17:Treg balance (decreased Th17 cells and increased Tregs) in the spleen was improved by propionate (Fig. 5D). These results suggest that propionate has therapeutic potential based on its anti-inflammatory activity.

### Propionate attenuates SS by inhibiting the STIM1-STING pathway

**Fig. 6:**
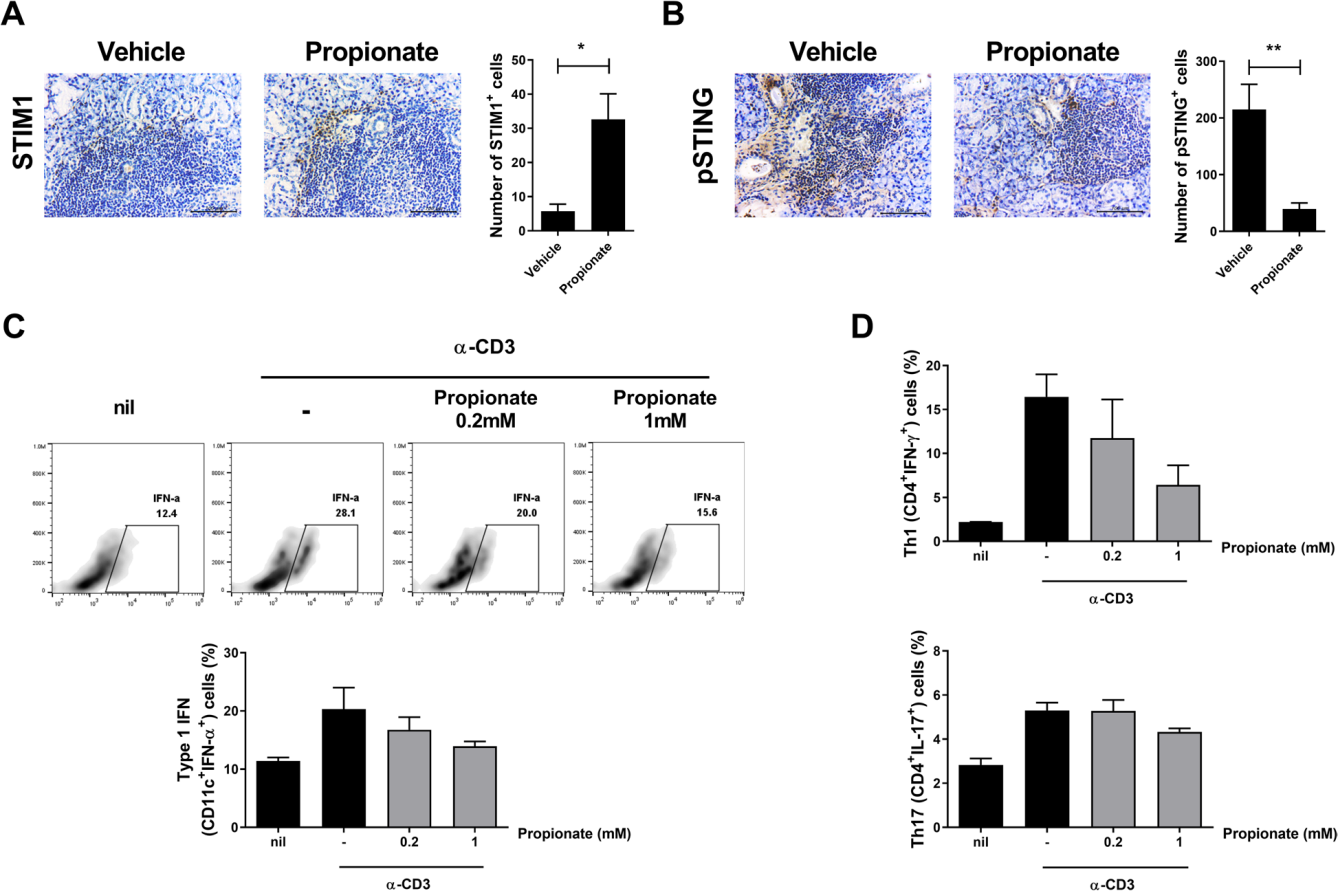
Propionate regulates expression of STIM1 and STING. Salivary gland tissue was stained for STIM1 and phospho-STING. **A** Representative images of STIM1 in the salivary gland and average numbers of STIM1-positive cells. **B** Representative images of phosph0-STING in the salivary gland and average numbers of phosphor-STING-positive cells. **C** Splenocytes were stimulated with anti-CD3 (0.5 µg/mL) in the absence or presence of propionate (0.2 or 1 mM) for 48 h and harvested for flow cytometry. FACS plots show percentages of type I IFN–positive cells. Bar graphs show average percentages of type I IFN–producing dendritic cells. **D** Percentages of Th1 (CD4^+^IFN-γ^+^) and Th17 (CD4^+^IL-17.^+^) cells. Data are means ± SD (*p < 0.05, ***p < 0.001)

STIM1 and phospho-STING expression were increased and decreased, respectively, by propionate (Fig. 6A, B). In addition, propionate decreased the number of type I IFN–producing cells (Fig. 6C, D). These data suggest that propionate ameliorates SS by inhibiting the production of type I IFN.

## Discussion

SS is characterized by immune cell infiltration and tissue destruction, leading to dry eyes and mouth. It is most commonly diagnosed in adults older than 40 years old and is more frequent in women. There is no cure for SS; treatment focuses on relieving symptoms [4]. In this study, we propose a microorganism (probiotic) and metabolite (SCFA) for the treatment of SS.

The gastrointestinal system and oral cavity are rich in microorganisms [43]. Probiotics reportedly have an immunomodulatory effect in inflammatory diseases [9, 13]. Because SS involves inflammation of the salivary and lacrimal glands, probiotics may have therapeutic potential. We reported that *L*. *acidophilus* improves intestinal inflammation in a model of DSS-induced IBD [16]. *L*. *acidophilus* decreases levels of inflammatory cytokines in the intestine and regulates the Th17:Treg balance. In addition, it has a therapeutic effect in lupus-prone mice by regulating the SIGNR3 pathway [17]. Manirarora et al. showed that lactobacilli have therapeutic potential for lupus by enhancing immunoregulation [44]. In this study, *L*. *acidophilus* ameliorated the development and progression of SS by decreasing the infiltration of inflammatory cytokine–producing cells into the salivary gland and improving the Th17:Treg balance. SIGNR3 regulates intestinal immunity [19]. *L*. *acidophilus* increases expression of SIGNR3. Our data suggest that *L*. *acidophilus* ameliorates SS by modulating SIGNR3 signaling.

Probiotics alter the microbial composition of the gut [45, 46]. Alcon-Giner et al. reported that induction of *Bifidobacterium* changes the composition of the gut microbiota [47]. Shi et al. showed that *Lactobacillus* supplementation restores the gut microbiota [48]. In this study, we found the therapeutic potential of administration of *L*. *acidophilus* through restored the gut microbiota in the SS animal model. Administration of *L*. *acidophilus* led to change *Bacteroides:Firmicutes* ratio as well as species richness and diversity. The species richness and diversity mean the number of species which reflect the gut microbiome health. Our data indicated that *L*. *acidophilus* ameliorate SS through altering gut environment. Furthermore, *L*. *acidophilus* increased the abundance of *Ruminococcaceae*, *Clostridiaceae*, and *Prevotellaceae* which are the propionate-producing bacterial taxa. Tedelind et al. reported that propionate has a therapeutic effect in inflammatory bowel disease [29]. Mizuno et al. showed that propionate ameliorates the severity of EAE in an animal model [49]. In this study, supplementation with propionate attenuated the development and progression of SS by decreasing the infiltration of inflammatory cytokine–producing cells into the salivary gland. Therefore, propionate ameliorates SS by regulating the Th17:Treg balance and thus has therapeutic potential for SS. Also, propionate is known to regulate insulin production. NOD mice used in this study often accompany diabetes with high blood glucose level. Increasing propionate by administration of *L*. *acidophilus* may be possible mechanism in regulating SS pathogenesity.

Type I IFN is produced by STING activation and is implicated in the pathogenesis of rheumatic diseases, including SLE, SS, and RA [50]. Type I IFNs have immunostimulatory properties, including the activation of B cells to produce autoantibodies [51]. Suppression of local type I IFN by the gut microbiota has been reported [40]. However, the role of the gut microbiota or metabolites in type I IFN expression is unclear. *L*. *acidophilus* and propionate increase expression of STIM1, a negative regulator of STING, and decrease expression of STING, an inducer of type I IFN. In addition, *L*. *acidophilus* and propionate decrease type I IFN expression in splenocytes. Our data suggest that *L*. *acidophilus* and propionate ameliorate SS by inhibiting the STIM1–STING–type I IFN axis. As we said, however, the role of microbiota and its metabolite in type I IFN production is still controversial. The effect of microbiota and metabolite varies by disease type, disease severity. It still requires further study to understand mechanism of regulating type I IFN production through microbiota and metabolite.

## Conclusions

Our results suggest that *L*. *acidophilus* and propionate have therapeutic potential for SS. Dysbiosis of the gut microbiota promotes SS by increasing the infiltration of lymphocytes into salivary glands and activating the STIM1–STING–type I IFN axis. Our findings demonstrate the therapeutic potential of *L*. *acidophilus* and propionate for SS.

## Supplementary Information

**Additional file 1: Supplementary Table 1.** List of antibodies usAQed in this study. **Supplementary Table 2.** List of primers used in this study.

## Data Availability

The authors confirm that the data supporting the findings of this study are available within the article. Gut microbiome analysis was deposited in the BioProject and Sequence Read Archive (SRA; https://www.ncbi.nlm.nih.gov/sra) under the accession numbers PRJNA863566.
