# Supplementary material for: Lactobacillus acidophilus and propionate attenuate Sjögren’s syndrome by modulating the STIM1-STING signaling pathway
Source: Cell Commun Signal. 2023 Jun 14;21:135. doi: 10.1186/s12964-023-01141-0 (PMC10265917; doi:10.1186/s12964-023-01141-0)
Supplement: Supplementary file 2 — Additional file 1: Supplementary Table 1. List of antibodies usAQed in this study. Supplementary Table 2. List of primers used in this study. [file 12964_2023_1141_MOESM1_ESM.docx]

**Supplementary Table 1. List of antibodies used in this study**

| **Target gene** | **Source** | **Identifier** | **Application** |
| --- | --- | --- | --- |
| IL-6 | Novus | NB600-1131 | Immunohistochemistry |
| IL-17 | Santa Cruz | SC-7927 | Immunohistochemistry |
| TNF-α | abcam | ab6671 | Immunohistochemistry |
| STIM1 | Invitrogen | MA1-19451 | Immunohistochemistry |
| Phospho-STING | Invitrogen | PA5-105674 | Immunohistochemistry |
| CD4 | Novus | NBP2-25191 | Confocal microscopy |
| CD25 | Novus | NB600-564 | Confocal microscopy |
| IL-17 | abcam | ab79056 | Confocal microscopy |
| FoxP3 | Novus | NB100-39002 | Confocal microscopy |
| Goat Anti-Rat IgG (H+L)-Alexa Fluor 488 | Invitrogen | A11006 | Confocal microscopy |
| Goat Anti-Rabbit IgG (H+L)-PE | Southernbiotech | 4050-09 | Confocal microscopy |
| Rat Anti-Mouse IgG1-APC | BD | 550874 | Confocal microscopy |
| DAPI | Invitrogen | D3571 | Confocal microscopy |
| CD4  (PerCP-Cyanine5.5) | eBioscience | 45-0042-82 | Flow Cytometry |
| CD19 (eFluor 450) | eBioscience | 48-0193-82 | Flow Cytometry |
| CD11c (APC) | BD | 550261 | Flow Cytometry |
| IFN-γ (APC) | BioLegend | 505810 | Flow Cytometry |
| IL-4 (PE) | BD Pharmingen | 554435 | Flow Cytometry |
| IL-17  (Alexa Fluor 488) | BioLegend | 506919 | Flow Cytometry |
| IFN-α (FITC) | R&D | 22100-3 | Flow Cytometry |

**Supplementary Table 2. List of primers used in this study**

| **Gene Name** | **Forward Primer** | **Reverse Primer** |
| --- | --- | --- |
| **Human**  **Β-actin** | CATGTACGTTGCTATCCAGGC | CTCCTTAATGTCACGCACGAT |
| **Human**  **SIGNR3** | GTGGGAAATAAAAGCTGTGGC | GAGTCTGTCGGAATCCAAGG |
| **Human**  **PD-L1** | TGGCATTTGCTGAACGCATTT | TGCAGCCAGGTCTAATTGTTTT |
| **Human**  **IDO** | GCTAAAGGCGCTGTTGGAAA | CCCTTCATACACCAGACCGT |
| **Human**  **IL-10** | TCAAGGCGCATGTGAACTCC | GATGTCAAACTCACTCATGGCT |
| **Mouse**  **Β-actin** | GAAATCGTGCGTGACATCAAAG | TGTAGTTTCATGGATGCCACAG |
| **Mouse**  **SIGNR3** | TCAAGAGTTTGGCAGAGTATACG | TTGTTCTGAACCTCTGAGCTG |
| **Mouse**  **PD-L1** | AAAGTCAATGCCCCATACCG | TTCTCTTCCCACTCACGGGT |
| **Mouse**  **IDO** | GACGGACTGAGAGGACACAG | GGCAGCACCTTTCGAACATC |
| **Mouse**  **IL-10** | GGCCCAGAAATCAAGGAGCA | AGAAATCGATGACAGCGCCT |
